# Comparison of Bond Strength Between Two Resin Cement Types and Additive Manufacturing or Cast Cobalt-Chromium Alloys

**DOI:** 10.7759/cureus.61041

**Published:** 2024-05-25

**Authors:** Koji Takahashi, So Koizumi, Katsura Ohashi, Tomotaro Nihei, Tetsutaro Yamaguchi

**Affiliations:** 1 Department of Orthodontics, School of Dentistry, Kanagawa Dental University, Yokosuka, JPN; 2 Department of Clinical Biomaterials, School of Dentistry, Kanagawa Dental University, Yokosuka, JPN

**Keywords:** orthodontics, rebonding, adhesion, resin luting cement, bond strength

## Abstract

Objective: To compare the bond strength of two types of resin cement to that of additive manufacturing (AM) or cast cobalt-chromium (Co-Cr) alloys.

Materials and methods: Two types of resin luting cement, composite resin and methyl methacrylate (MMA), were bonded to AM or cast Co-Cr alloys, and shear bond tests were performed after seven days of storage in distilled water at 37°C. Co-Cr alloy adhesive elements AM to the enamel surface of the labial aspect of a bovine mandibular central incisor crown were bonded with two types of resin luting cement and subjected to 1,000 cycles of storage in water for one day and 28 days or thermal cycling, followed by shear bonding tests. Residual cement on the metal and enamel surfaces after the bonding tests was evaluated using an optical microscope. The normality of the results was evaluated using statistical software Statcel4, analysis of variance, or Kruskal-Wallis test, depending on normality, and multiple comparison tests were performed using the Tukey-Kramer or Steel-Dwass tests.

Results: After one day, the shear bond strength (SBS) was 25.9 MPa for Panavia V5 (PV; Kuraray Noritake Dental Corporation, Niigata, Japan) and 23.5 MPa for Super-Bond (SB; Sun Medical Corporation, Shiga, Japan), with no significant difference between the two cement types (P > 0.05). After 28 days, the SBS decreased to 4.1 MPa for PV and 6.7 MPa for SB, showing a significant difference between the two cements (P < 0.05). Following 1,000 thermal cycles, the SBS was 2.0 MPa for PV and 5.6 MPa for SB, with SB exhibiting a significantly higher value (P < 0.05). The adhesive strength was significantly lower after 28 days of storage and thermal cycling compared to after one day of storage (P < 0.05). The Co-Cr alloy exhibited more residual cement on the enamel surface due to interfacial fracture with the resin cement. The Co-Cr alloy showed more residual cement on the enamel surface due to interfacial fracture with the resin cement.

Conclusion: MMA-based resin cement showed optimal bond strength and may be suitable for clinical use in computer-aided design (CAD)/computer-aided manufacturing (CAM) orthodontic appliances.

## Introduction

Cast prosthetics are commonly used for crown restorations and dentures in dentistry, whereas wire fixation devices are used in orthodontics. Recently, metal 3D printers for additive manufacturing (AM) have attracted attention in dentistry [1]. Cobalt-chromium (Co-Cr) alloys are used for fixed orthodontic appliances, most attached by direct bonding. Fixed orthodontic appliances include direct bonding systems (DBS) to enamel and orthodontic bands. Appliances such as lingual arches and Nance's holdings, which are conventionally fabricated with orthodontic bands, can be fabricated using a metal 3D printer, simplifying fabrication and significantly reducing chair time [2]. George Newman [3] developed DBS in the mid-1960s. Miura et al. [4] developed a technique to bond polycarbonate plastic brackets to phosphate-etched enamel using a restorative filling material in the early 1970s. The adhesive strength of fixed orthodontic appliances to enamel surfaces is controversial in orthodontic treatment. Ortendahl and Thilander [5] reported a minimum shear bond strength of 4 MPa, Reynolds et al. [6] of 5.9-7.8 MPa, and McCourt et al. [7] reported a minimum of 10 MPa. Adhesion theory is not well understood, and bracket detachment and enamel damage may occur with improper manipulation of the bond. Bracket detachment can be attributed to improper bonding procedures [8], salivary contamination during bonding [9], and excessive occlusal forces after bonding [10]. The appliance is removed at the end of orthodontic treatment and is not fixed permanently; however, the bond strength must withstand continuous orthodontic forces applied throughout the treatment period. Changes in the bond strength of cast Co-Cr alloy prosthetics over time have been reported [11]; however, the bond strength of metal 3D-printed orthodontic devices under conditions simulating the intraoral environment is unknown.

We aimed to compare the bond strength of two types of resin cement to that of additive manufacturing or cast Co-Cr alloys and investigate the bond strength to enamel in clinical use.

## Materials and methods

Materials　

Super-Bond (SB) (Sun Medical Corporation, Shiga, Japan) and Panavia V5 (PV) (Kuraray Noritake Dental Corporation, Niigata, Japan) were used as resin cement. Clearfill Ceramic Primer Plus (CP) (Kuraray Noritake Dental Corporation) was used for metal surface treatment, in addition to Tooth Primer (TP) (Kuraray Noritake Dental Corporation) and 65% phosphoric acid etching (E65%) (surface treatment agent red, Sun Medical Corporation) (Tables 1, 2). Samples were powder-fabricated Co-Cr alloys (3D) (SP2; EOS, Kreiling, Germany) for AM using a metal 3D printer (EOSINT M 270 Dental; EOS) and cast Co-Cr alloys (CA) (Biogil f; Dentsply Sirona, Tokyo, Japan).

**Table 1:** Super-Bond (Sun Medical Corporation) MMA: methyl methacrylate, 4-META: 4-methacryloyloxyethoxycarbonylphthalic anhydride, PMMA: polymethylmethacrylate.

Components	Properties	Description	Lot. No.	Abbreviation
Quick Monomer Liquid	Liquid	MMA, 4-META, etc.	EW1	QM
Catalyst V	Liquid	Tri-n-butyl boron partial oxide, etc.	FR33R	CV
Polymer Powder	Powder	PMMA, contains sodium fluoride	EV11	―
Surface Treatment Agent Red	Liquid	Phosphoric acid, water, etc.	ET２	―
Teeth Primer	Liquid	4-META, water, acetone, etc.	EW1	TP

**Table 2:** Panavia V5 (Kuraray Noritake Dental Corporation) Bis-GMA: bisphenol A-glycidyl methacrylate, TEGDMA: triethylene glycol dimethacrylate, MDP: 10-methacryloyloxydecyl dihydrogen phosphate, HEMA: hydroxyethylmethacrylate.

Components	Properties	Description	Lot. No.	Abbreviation
Paste (universal) A paste	Paste	Monomer (Bis-GMA, TEGDMA, other methacrylate monomers), filler (surface treated basium glass, surface treated fluoroaluminosilicate glass, surface treated alumina microfiller)	AD0175	PA
Paste (universal) B Paste	Paste	Monomer (Bis-GMA, other methacrylic acid monomer), filler (surface-treated barium glass, surface-treated alumina microfiller), photo polymerization catalyst, polymerization accelerator, coloring agent, etc.	AD0175	PB
Tooth Primer	Liquid	Monomer (MDP, HEMA, other methacrylic acid monomer), purified water, polymerization accelerator, others	850096	TP
Clearfill Ceramic Primer Plus	Liquid	Silane coupling agent, monomer (MDP), ethanol	890067	CP
Surface Treatment Agent Red	Liquid	Phosphoric acid, water, etc.	EM1	―

Adhesion of resin cement to Co-Cr alloys

The surface of a metal sample was protruded by 1.0 mm, the bottom was embedded in resin, and metal surface was polished flat with water-resistant abrasive paper of up to #600. After ultrasonic cleaning for 30 minutes, the sample was subjected to alumina sandblasting (particle size: 250 μm, blasting pressure: 0.2 MPa) at a distance of 10 mm for 10 s. Subsequently, another 30 min ultrasonic cleaning was performed. Surface of the metal specimen meant to be filled with PV was coated with CP and dried. Mending tape (3M Japan Co., Ltd., Tokyo, Japan) with a φ3.0 mm hole was applied to the adherend surface to define the bonding area, and each resin cement was placed in a brass mold with a height of 3.0 mm, an inner diameter of 5.0 mm and an outer diameter of 7.0 mm. SB was loaded with polymer powder F3 brushes using the brush stacking method, and a load of 1 kg was applied from the top surface via a glass slide for 7 min. PV was loaded directly into the mold from a syringe under a 1 kg load for 1 min and irradiated from the top surface for 20 s using a light illuminator (G-Light Prima-II; GC Corporation, Tokyo, Japan). Samples were stored in deionized water at 37 ° C for seven days, and shear adhesion tests were performed using a small table-top testing machine (EZ test; Shimadzu Corporation, Kyoto, Japan) at a crosshead speed of 1.0 mm/min. The number of specimens in each group was 12. After the shear bond test, the fracture surface was observed using a stereomicroscope (SZ61; Olympus Corporation, Tokyo, Japan), and the state of residual resin cement on the metal surface was evaluated using the adhesive residual index (ARI) (Table 3).

**Table 3:** Classification of Resin Cement Residue on the Metal Surface after the Shear Bond Test (ARI) ARI: adhesive residual index.

Class	Fracture surface condition
0	No resin cement residue on the metal surface
1	Less than half of the resin cement remaining on the metal surface
2	More than half of the resin cement remaining on the metal surface
3	All resin cement remaining on the metal surface

Adhesion of resin cement to Co-Cr Alloy and enamel

Enamel on the labial side of the crown of a bovine mandibular central incisor was used as the adherend. The crown was embedded in resin with the labial side protruding 1.0 mm, and the enamel surface was polished flat with water-resistant abrasive paper to #600, rinsed, and dried. The tooth surface was left for 30 s after E65% application, rinsed for 30 s, and dried for 10 s. Regarding tooth surface treatment, the tooth surface was left for 20 s after TP application, rinsed for 30 s, and dried for 10 s. After treatment, a mending tape with φ3.0 mm was applied to define the bonding area, and CP was applied to the bonding surfaces of the Co-Cr alloys to be bonded by PV and allowed to dry. Co-Cr alloy glue specimens with a diameter of 5.0 mm were glued with SB using a polymer powder brush stacking method with F3, and a load of 1 kg was applied for 5 min. PV was applied directly from a syringe, and a load of 1 kg was applied for five min, followed by irradiation from the top surface for 10 s using a light illuminator. After gluing, each specimen was divided into two groups: one kept in deionized water at 37°C for one day and 28 days, and the other was immersed in a water bath at 5°C and 55°C for 40 s each for 1,000 thermal cycles. Subsequently, shear adhesion tests were conducted using the EZ test, a small table-top tester, at a crosshead speed of 1.0 mm/min. The number of specimens in each group was 12. The remaining resin cement condition on the enamel surface was evaluated by the ARI (Table 4).

**Table 4:** Classification of Resin Cement Residue on the Enamel Surface after the Shear Bond Test (ARI) ARI: adhesive residual index.

Class	Fracture surface condition
0	No resin cement residue on the enamel surface
1	Less than half of the resin cement remaining on the enamel surface
2	More than half of the resin cement remaining on the enamel surface
3	All resin cement remaining on the enamel surface

Surface analysis of Co-Cr alloys

Sample surfaces were analyzed using a scanning X-ray photoelectron spectrometer (PHI 5000 VersaProbe III; ULVAC Phi Corporation, Kanagawa, Japan), with an AlKα line of 1486.6 eV, an X-ray beam diameter of 100 μm, a photoelectron extraction angle of 45° to the sample normal, neutralization, and sputtering with the monomer Ar+. The thicknesses of the oxide films of the cast and AM Co-Cr alloys were measured under these conditions.The surface analysis, including measurement and interpretation, was conducted by a single researcher. The measurements were performed by an external service provider to ensure accuracy and consistency. To minimize bias, the measurement locations on the samples were chosen to avoid the edges and any contaminated areas, ensuring that clean, representative regions were analyzed. Additionally, the researcher performing the analysis was blinded to the specific history of the samples.

Statistical analysis

The adhesion results of resin cement to Co-Cr alloys were tested for normality using statistical software (Statcel4; OMS Publishing, Tokyo, Japan), and confirmed using analysis of variance (ANOVA). Results of the adhesion of resin cement to Co-Cr alloys and enamel were analyzed by initially applying a normality test and subsequently using the Kruskal-Wallis test. Multiple comparisons were conducted using the Steel-Dwass test. Statistical significance was set at p=0.05 for all analyses.

## Results

Adhesion of resin cement to Co-Cr alloys

**Figure 1:**
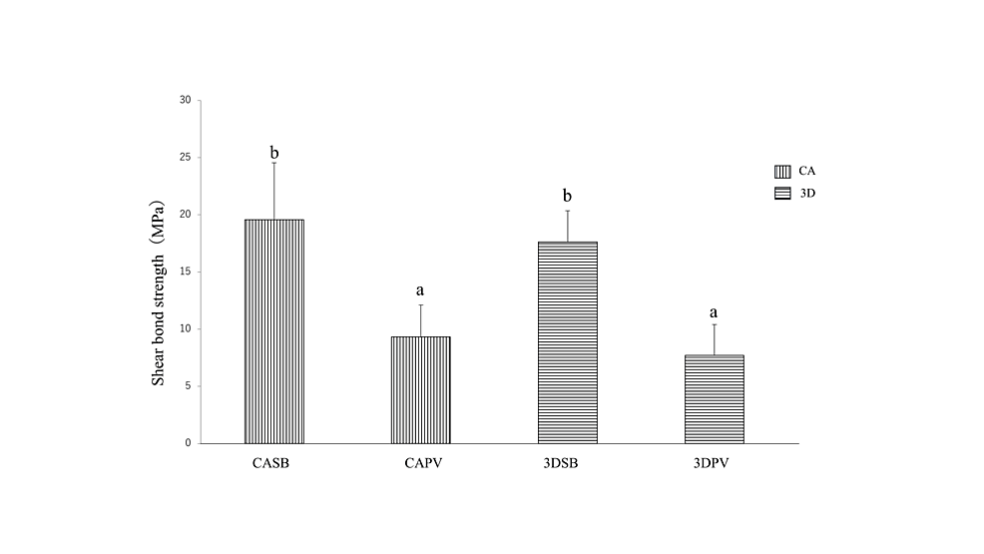
Shear bond strength of each resin cement for additive manufacturing and cast cobalt-chromium alloys The values of the two groups bonded with SB for the additive manufacturing and cast cobalt-chromium alloys were significantly higher than those bonded with PV (P<0.05). Different letters: significant difference between requirement groups. CA: cast cobalt-chromium alloys, 3D: additive manufacturing cobalt-chromium alloys, CASB: adhesion of cast cobalt-chromium alloys to Super-Bond, CAPV: adhesion of cast cobalt-chromium alloys to Panavia V5, 3DSB: adhesion of additive manufacturing cobalt-chromium alloys to Super-Bond, 3DPV: adhesion of additive manufacturing cobalt-chromium alloys to Panavia V5.

The shear bond strength (SBS) of the resin cement types to AM and cast Co-Cr alloys was measured after seven days of bonding. Results showed that the SBS of resin cement to the AM Co-Cr alloy was 7.7 MPa for PV and 17.6 MPa for SB, while the SBS to the cast Co-Cr alloy was 9.3 MPa for PV and 19.6 MPa for SB. The SBSs of the two groups of AM and cast Co-Cr alloys bonded with SB were significantly higher than those of the two groups bonded with PV (P < 0.05). No significant difference was observed between the two groups bonded with SB and PV in AM and cast Co-Cr alloys (P > 0.05). All ARI scores indicated interface fracture, with a score of 0 for both SB and PV (Table 5). Figure 1 shows these SBS values.

**Table 5:** ARI scores after bonding of each resin cement to cobalt-chromium alloys (n=12) ARI: adhesive residual index. ARI = 0: no adhesive, ARI = 1: less than 50%, ARI = 2: more than 50%, ARI = 3: 100% adhesive. CASB: adhesion of cast cobalt-chromium alloys to Super-Bond, CAPV: adhesion of cast cobalt-chromium alloys to Panavia V5, 3DSB: adhesion of additive manufacturing cobalt-chromium alloys to Super-Bond, 3DPV: adhesion of additive manufacturing cobalt-chromium alloys to Panavia V5.

Condition of fracture surface after the adhesion test												
Sample No.	1	2	3	4	5	6	7	8	9	10	11	12
CASB	0	0	0	0	0	0	0	0	0	0	0	0
CAPV	0	0	0	0	0	0	0	0	0	0	0	0
3DSB	0	0	0	0	0	0	0	0	0	0	0	0
3DPV	0	0	0	0	0	0	0	0	0	0	0	0

Adhesion of resin cement to Co-Cr alloy and enamel

**Figure 2:**
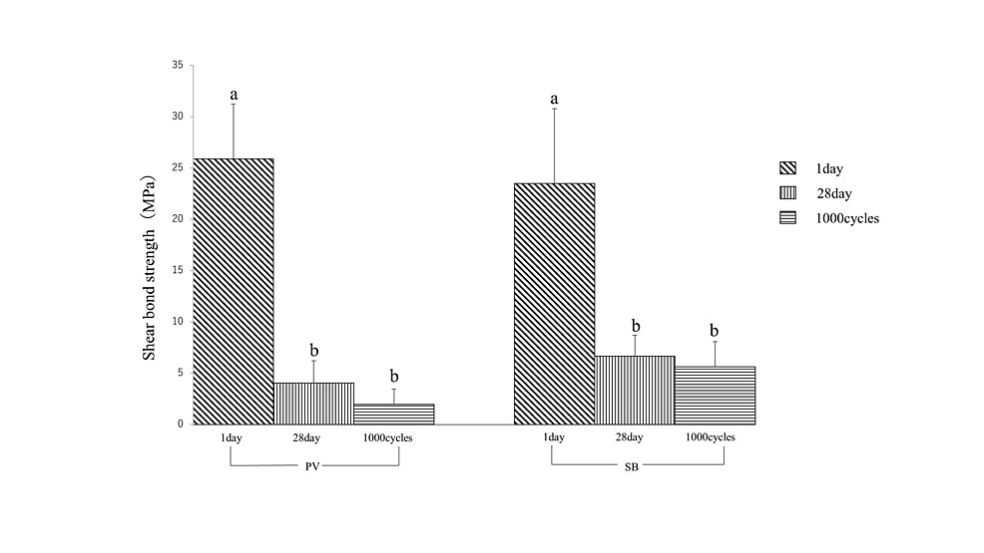
Shear bond strength of additive manufacturing cobalt-chromium alloy to enamel No significant difference was observed in shear bond strength between the two cement types after one day of storage (P > 0.05). A significant difference was observed between the two cement types after 28 days of storage (P<0.05). The values were significantly higher for SB than for PV after thermal cycle loading (P<0.05). Different letters: significant difference between requirement groups. PV: adhesion of additive manufacturing cobalt-chromium alloy and enamel to Panavia V5, SB: adhesion of additive manufacturing cobalt-chromium alloy and enamel to Super-Bond.

The SBS of Co-Cr alloys bonded to enamel was evaluated after one day, 28 days of storage, and 1,000 thermal cycles. After one day, the SBS was 25.9 MPa for PV and 23.5 MPa for SB, with no significant difference between the two cement types (P > 0.05). After 28 days, the SBS decreased to 4.1 MPa for PV and 6.7 MPa for SB, showing a significant difference between the two cements (P < 0.05). Following 1,000 thermal cycles, the SBS was 2.0 MPa for PV and 5.6 MPa for SB, with SB exhibiting a significantly higher value (P < 0.05). The adhesive strength was significantly lower after 28 days of storage and thermal cycling compared to after one day of storage (P < 0.05). Most ARI scores were 2 and 3 for both SB and PV, indicating residual resin cement on the enamel (Tables 6, 7). Figure 2 shows these SBS values.

**Table 6:** ARI scores after bonding cobalt-chromium alloys to enamel and Super-Bond (n=12) ARI: adhesive residual index. ARI = 0: no adhesive, ARI = 1: less than 50%, ARI = 2: more than 50%, ARI = 3: 100% adhesive.

Condition of fracture surface after the adhesion test												
Sample No.	1	2	3	4	5	6	7	8	9	10	11	12
1 day	2	3	3	3	3	3	3	3	2	3	3	3
28 days	3	3	3	3	3	3	3	3	2	3	3	3
thermal cycle	2	3	3	3	3	2	3	3	3	3	3	3

**Table 7:** ARI scores after bonding cobalt-chromium alloys to enamel and Panavia V5 (n=12) ARI: adhesive residual index. ARI = 0: no adhesive, ARI = 1: less than 50%, ARI = 2: more than 50%, ARI = 3: 100% adhesive.

Condition of fracture surface after the adhesion test												
Sample No.	1	2	3	4	5	6	7	8	9	10	11	12
1 day	2	2	3	2	2	2	3	1	2	2	3	2
28 days	2	3	3	3	3	3	3	3	3	3	3	2
thermal cycle	3	3	3	3	3	3	3	2	3	2	1	3

Surface analysis of Co-Cr alloys

**Figure 3:**
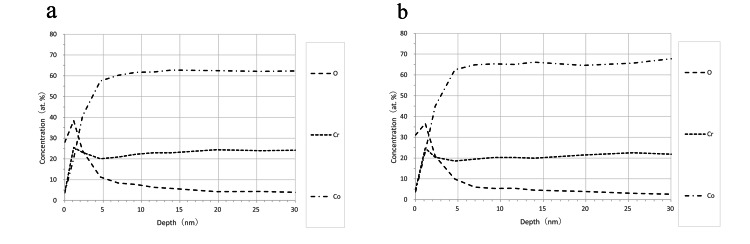
Atomic (compositional) analysis of cobalt-chromium alloys by depth using X-ray photoelectron spectroscopy The oxide film thickness of (a) cast cobalt-chromium alloys and (b) additive manufacturing cobalt-chromium alloys was determined as the depth at which the O concentration is half of the base concentration (0% in this case) and the highest concentration. The oxide film thickness of all samples was approximately 3 nm, and no significant difference was observed between the samples. O: oxygen, Cr: chromium, Co: cobalt. at: atomic.

The thickness of the oxide film was measured for both cast and additive manufacturing Co-Cr alloys. Results indicated that the oxide film thickness was 3.2 nm for the cast alloy and 2.9 nm for the additive manufacturing alloy, with no significant difference observed between the two samples (P > 0.05). Figure 3 shows a visual representation of these measurements.

Based on the obtained results, a post-hoc power analysis was conducted using G*Power Version 3.1.9.7 (Franz Faul, Kiel University, Kiel, Germany). The statistical power was found to be more than 98%. It is important to note that this study was exploratory in nature and was conducted with a sample size of n=12.

## Discussion

This study examined the SBS of two types of cement to AM and cast Co-Cr alloys. The SBS of resin cement to the Co-Cr alloy was significantly higher for SB than PV, regardless of the metal fabrication method. The SB primer, a MMA-based resin cement, contains 4-methacryloyloxyethoxycarbonylphthalic anhydride (4-META), and the composite resin cement contains 10-methacryloyloxydecyl dihydrogen phosphate (MDP), suggesting that the oxide film on surface of the non-metallic Co-Cr alloy may be responsible for adhesion to the cement [12]. The oxide film formed on the cast surfaces and AM Co-Cr alloys may explain the similar adhesive strengths observed, regardless of the processing method. When exposed to air, the Co-Cr alloy surface immediately reacts with oxygen to form an oxide layer of 1-3 nm thickness [13,14]. A chromium oxide or passive film layer is easily formed on the Co-Cr metal surface. The functional monomers contained in each adhesive primer have an affinity with the chromium oxide layer [15], and the oxide layer reacts with water in the air, covering the surface with hydroxyl groups (-OH) and bonding with the hydrophilic groups of the adhesive monomers, 4-META and MDP [16]. However, the composite resin cement was significantly lower than the MMA resin cement because the silane coupling agent, a primer for composite resin cement that normally acts on MDP to promote adhesion to metal, may have inhibited the original function of MDP instead [17]. The MMA-based resin cement may have formed a polymer mesh structure at the interface between the Co-Cr alloy and the resin cement [18], resulting in higher bond strength than composite resin cement. ARI score was only 0, indicating the lack of resin cement residue on the metal surface and fracture of the interface between the Co-Cr alloy and the resin cement.

The SBS between the Co-Cr alloy and enamel showed no significant difference between SB and PV after one day of bonding. However, the SBS of SB was significantly higher than that of PV after 28 days of storage in water and 1,000 thermal cycles, regardless of the metal processing method; likely due to the demineralization of enamel surface by etching and the formation of a microcavity structure, which allowed 4-META to penetrate and harden, resulting in a mechanically stronger anchorage effect [19]. MDP, the adhesive primer of PV, likely reacted with calcium ions that leached from dentin apatite to generate calcium salts [20], providing sufficient bond strength to the cement. PV demonstrated similar adhesive strength as SB after one day of storage; however, the adhesive strength decreased after 28 days of storage in water and after thermal cycling. The silane-treated layer on the surface of the inorganic filler of the composite resin cement was likely hydrolyzed [21], resulting in decreased bond strength [22]. Water may weaken mechanical properties by penetrating the matrix and reducing the frictional force between polymer chains, causing softening of the polymer resin components [23]. Thermal cycling may cause shrinkage and expansion owing to the difference in thermal expansion coefficients of enamel, resin cement, and Co-Cr alloy [24]. ARI score is not limited to cement type and bonding conditions. Score 2, representing more than half of the resin cement remaining on the enamel surface, and score 3, representing the resin cement remaining, were mostly reported, indicating low adhesion to metal and high adhesion to enamel. Acidic monomers have excellent permeability and diffusivity in demineralized enamel [25,26], and the adhesive monomers 4-META and MDP were more likely to penetrate demineralized enamel treated with tooth surfaces, mechanically bond with the cured resin layer, and react with enamel hydroxyapatite. Resin cements were more adhesive to enamel than the cobalt-chromium alloys because the resin monomers penetrated the demineralized enamel and reacted with the enamel hydroxyapatite. Clinically, the enamel damage risk is reduced when more cement remains in the enamel during debonding; however, the chairside time required for cement removal is longer.

The bond strength between fixed orthodontic appliances and adhesives can be improved by redesigning the bonding surface of the appliance [27] and sandblasting the bonding surface [28]. Fixed orthodontic appliances are worn for several years; therefore, evaluating the durability of adhesive materials using in vitro adhesion tests is necessary. The SBS after 1,000 cycles of thermal cycling between 5-55°C was significantly lower than that after one day and 28 days of storage in water for both SB and PV. PV after thermal cycling was 1.9 MPa, which was lower than the optimal bond strength of 4 MPa reported by Ortendahl and Thilander. The SB was 5.6 MPa, suggesting adequate strength for clinical use in bonding orthodontic appliances. Retief et al. [29] reported that the bond load should not exceed 14 MPa to avoid enamel damage, suggesting that the SBS of SB may withstand normal orthodontic forces and prevent enamel damage during debonding. Although this study was able to investigate the bond strength of two types of resin luting cements, further research is necessary to evaluate enamel delamination after shear bond tests using scanning electron microscopy (SEM). This will provide a more comprehensive understanding of the effects of these adhesives on enamel integrity.

## Conclusions

MMA-based cement showed higher bond strength than composite resin-based cement, regardless of metal fabrication method. This MMA-based resin cement may provide clinically adequate bond strength for fixed computer-aided design (CAD)/computer-aided manufacturing (CAM) orthodontic appliances compared to composite resin-based cement. Although the present study was able to examine the bond strength after a certain period of time under conditions that mimic the oral environment, more clinical bonding studies are needed to investigate the in vivo performance of MMA-based resin cements and composite resin cements and to determine appropriate bond strength while preventing enamel delamination during debonding.
